# Preparedness League of American Dentists

**Published:** 1919-02

**Authors:** 


### PREPAREDNESS LEAGUE OF AMERICAN DENTISTS

### EXPANSION OF THE LEAGUE

---

The League has made a permanent place for itself. It has proven its usefulness to the dental profession and its necessity to the welfare of humanity. Its field of operation reaches wherever organized dentistry exists, and its public service may be felt to the extreme limits of civilization. Such is a brief outline of the field of our future endeavors.

In expanding the activities of the League, we desire, however, to lay down the following unvarying rules and principles that any misconception of our aims may not occur:

1. The principle of the League is Service to Humanity.

2. Politics or promotion of personal interests *shall not be tolerated.*

3. It is essentially an organization for public health service.

4. Its activities shall not conflict with other dental organizations in any way.

5. Its object shall be the co-ordination of all professional and public agencies which may thereby render better service for the common welfare of humanity.

6. No officer or member of the League shall receive pay for services unless of such character as to be justifiable, but not without proper action by a quorum.

